# Drug sensitivity testing of patient-derived bone sarcomas identifies selective kinase inhibitors for patients with refractory disease

**DOI:** 10.1007/s12094-026-04261-4

**Published:** 2026-04-13

**Authors:** Christina Linder-Stragliotto, Panagiotis Tsagkozis, Swapnil Potdar, Carolina Alicia Brodin, Bertha Alicia Brodin

**Affiliations:** 1 Department of Molecular Medicine and Surgery, Karolinska Institutet, Stockholm, Sweden; 2 Institute for Molecular Medicine, Helsinki, Finland; 3 Karolinska University Hospital, Southern Hospital (Södersjukhuset), Dept Cardiology and Intensive Care, Stockholm, Sweden; 4 Present Address: Boston Consulting Group, Stockholm, Sweden; 5 Department of Applied Physics, Royal Institute of Technology (KTH), Stockholm, Sweden

**Keywords:** Precision medicine, Bone sarcoma, Drug sensitivity testing, Ewing´s sarcoma, Osteosarcoma

## Abstract

**Objective:**

This study aimed to investigate the feasibility and predictive value in performing ex vivo drug sensitivity testing on bone sarcomas from patients with refractory disease and identify agents with potential therapeutic benefit.

**Methods:**

A cohort of 7 osteosarcomas (OS) and 4 Ewing sarcoma (ES) patient-derived cells (PDCs) were screened against a library of oncological drugs, gene panel sequencing and mRNA expression arrays. Drug responses were correlated to the molecular characteristics of specific sarcoma subtypes and patient response to therapy.

**Results:**

OS PDCs showed heterogeneous drug sensitivity, with observed responses for mTOR, PKC, MAPK and CDK inhibitors that correlated with the histological subtype and genotype of the primary tumor. Two of 4 ES PDCs displayed morphological dichotomy as separate *Ews-Fli1* positive spheroid and adherent populations. These ES PDCs showed comparable responses to most of the oncological drugs but differential sensitivity to rapalogs and SMAC-mimetics. In both, OS and ES, the drug sensitivity of PDCs correlated to the patient response to chemotherapy.

**Conclusion:**

Drug sensitivity testing of PDCs from bone sarcomas is a valuable tool for a rapid identification of potential treatments, in otherwise genetically complex group of tumors where genome-based assays are difficult to implement. Clones with different phenotypes can outgrow in PDC cultures and can represent clonal evolution that give raise to tumor recurrence.

**Supplementary Information:**

The online version contains supplementary material available at https://doi.org/10.1007/s12094-026-04261-4.

## Introduction

Primary bone sarcomas are heterogeneous tumors comprising less than 2% of all adult cancers. Osteosarcoma (OS) and Ewing sarcoma (ES) are the most common bone sarcomas in adolescents and young adults and represent 5% of all newly diagnosed cancers in this population [1, 2].

Osteosarcomas can be diagnosed as low-grade tumors arising from the medullary cavity of the bone, as periosteal intermediate-grade tumors located in the surface of the metaphysis of long bone, or as the conventional high-grade intramedullary tumors producing osteoid [2]. Histologically, they are composed of neoplastic and pleomorphic mesenchymal cells in intimate cross talk with their bone microenvironment, composed of osteoblasts, osteoclasts, and cells of the immune system. This microenvironment impacts the tumor response to treatment. Patients affected by osteosarcoma undergo surgery combined with or adjuvant and/or postoperative chemotherapy. For example, the EURAMOS 1 chemotherapy regimen used for the treatment of resectable OS has been thoroughly evaluated in large patient cohorts. It consists of two preoperative cycles of methotrexate, adriamycin, and cis-platin (MAP) followed by additional postoperative MAP or just MA cycles with the possibility of the addition of ifosfamide in poor responders [3, 4].

A hallmark for OS is genetic instability. The tumors are aneuploid and display a high rate of chromosomal aberrations [5–7], gene mutations most frequent in the TP53, RB1, and ATRX genes [8]. Chromothripsis, defined as a cluster of rearrangements such as gene amplifications, develops in localized chromosomal regions [9] and contributes to genome destabilization [10]. In spite of this genomic complexity, osteosarcomas can be stratified into four functional groups based on gene expression patterns such as immune activated, immune suppressed, osteosarcomas with dominant autologous recombination deficiency, and *cMyc* driven [11]. Because of their genomic complexity, oncologic treatments based on specific gene alterations are difficult to implement. Functional assays such as drug screening on patient tumor cell models are emerging as robust models for precision oncology [12–14].

Ewing sarcoma is the second most common bone sarcoma that, in contrast to osteosarcoma, is driven by a single recurrent chromosomal translocation between *EWSR1* and an *ETS* family member, most commonly *FLI1*, leading to the aberrant expression of EWS-FLI1 [15]. The fusion protein is a dual transcription factor that binds target genes with ETS-like binding motifs. ES affects pediatric and adolescent populations, is genetically stable, and displays a low gene mutation rate. Most frequently mutated genes are *STAG2* (15–22%), *TP53* (6–7%), and *CDKN2A* (12–28%) [16–18]. Despite this genomic stability, *Ews-Fli1* carrying tumors are composed of metabolically heterogeneous populations as demonstrated by spatial single cell RNA sequencing on patient-derived xenografts [19].

Localized ES is treated with alternating cycles of vincristine–doxorubicin–cyclophosphamide and ifosfamide–etoposide (VDC/IE) as induction chemotherapy, and the same regimen is used for consolidation chemotherapy.

With the aim to identify oncologic drugs as potential treatments for patients with advanced sarcomas, we previously reported the feasibility and clinical value of performing drug screening assays on patient-derived translocation-carrying soft tissue sarcomas [20]. We now present our experience in performing drug sensitivity testing on patient-derived bone sarcomas and show its value in identifying specific drug responses that are associated with the genomic aberrations and histological subtype of each osteosarcoma. In addition, we report intra-tumor heterogeneity in *Ews-Fli1* PDCs defined by morphology and drug response.

## Materials and methods

### Establishment of osteosarcoma and Ewing’s sarcoma patient-derived cells (PDC)

Patient-derived cells were obtained from surgical specimens or fine needle aspiration biopsies. Osteosarcoma cells were dissociated from tissue specimens by enzymatic digestion using a 0.1% collagenase for 1–2 h at 37 °C under gentle agitation. The cell mixture was then passed through a 100 μm mesh, and thereafter centrifuged and resuspended in Dulbecco’s modified Eagle’s medium/Ham’s nutrient mixture F12 (DMEM/F12) supplemented with 15% fetal bovine serum (FBS), non-essential amino acids, penicillin, and streptomycin. Ewing sarcoma cells obtained from FNAs were grown on fibronectin-coated plates using the same culturing medium. Exponentially growing patient-derived cells (PDC) were established within a range from 2 days to 18 weeks depending on the biopsy size, cell viability at biopsy collection, and histological subtype. A summary of the bone sarcoma PDC characterization is shown in Supplementary Information 3 and Supplementary Information 1.

### Drug sensitivity testing

Drug sensitivity testing was performed using the oncology drug library from the Institute for Molecular Medicine Finland (FIMM) as previously described [20]. It consists of 525 approved and experimental drugs dispensed in 380-well plates at five different concentrations within a range of 1 to 10,000 nM each. Approximately 2000 PDCs in a total volume of 25 μL were dispensed to each well using an automatic dispenser (Multidrop Combi Reagent Dispenser, Thermo Fisher Scientific, Maltham, MA, USA). The plates were subsequently incubated for 72 h at 37 °C and in 5% CO_2_ in a humidified environment. Cell viability was determined using CellTiter-Glo (CTG) Luminescent cell viability assay (Promega, Madison, Wisconsin, USA) measuring the total ATP levels in living cells and luminescence was measured using an SpectraMax iD5e Multi-mode Microplate Reade (Molecular Devices). Drug sensitivity scores (DSS) were calculated using the drug screening data analysis tool Breeze [21]. (https://breeze.fimm.fi/v2/database.php). The DSS values were further normalized against the DSS values of healthy muscle tissue to obtain the selective drug sensitivity score (sDSS).

DST data sets were analyzed using the free, web-based software tool Morpheus, https://software.broadinstitute.org/morpheus.

### Determination of Ews-Fli1 fusion protein in Ewing sarcoma PDCs by proximity ligation assay (PLA)

First passage Ewing sarcoma PDCs were cultured in chamber slides for 24 hours, washed twice with phosphate buffered saline (PBS) and fixed for 15 min in 4% paraformaldehyde, and thereafter permeabilized with 0.1% TritonX100 for 15 min. The PLA was performed according to the in situ fluorescence PLA protocol (Sigma-Aldrich) using an antibody recognizing the N-terminus of Ews (mouse monoclonal antibody C9, Santa Cruz Biotechnology) and the other recognizing the C-terminus of the Fli-1 (rabbit monoclonal antibody AbCam 15289). Species-specific antibodies conjugated with DNA oligonucleotides were then used to recognize the primary antibodies allowing to form a circular probe provided both primary antibodies are in the proximity (i.e., anti Ews and Fli1). The DNA probe is subsequently amplified using DNA polymerase and a fluorescent-labeled deoxynucleotide in the amplification reaction allowing the visualization of the fusion protein in the UV-light range using fluorescent microscopy.

### Cancer driver gene expression

mRNA was isolated using Allprep (Qiagen, Hilden, Germany) according to the manufacturer’s instructions. The quality and concentration of the mRNA was determined using the Agilent bioanalyser (Sta Clara CA, USA). Quantitative gene expression of 180-cancer driver genes was performed using PCR-arrays (RealTimePrimers, Elkins Park PA, USA) and AzuraQuant™ Green Fast qRT-PCR green master mix (Azura Genomics, Raynham, MA, USA). Analysis of gene expression was performed by calculations according to the Livak method in which ΔΔCT of the patient tumor (referred to as Log_2_ Fold change) was calculated in relation to the ΔCT values of normal muscle cells.

### Target enrichment and next-generation sequencing

Target enrichment was performed with the HaloPlex target enrichment system (Agilent Technologies), as previously described. A custom gene panel was designed, covering the coding regions of the 16 target genes including: *STAG2, CDKN2A, TP53, PTEN, NF1, BRAF, CTNNB1, SRC, RB1, CDH1, P14KA, RICTOR, EZH2, BCOR*, and *ARID1.* The target genes were captured by 6063 probes, covering a total region of approximately 76 kb. The quality and molarity of the sequencing libraries were assessed on a 2200 TapeStation instrument using a D1000 Screen tape (Agilent Technologies). The enriched and barcoded targets were then sequenced on a MiniSeq or NextSeq instrument (Illumina).

## Results

### Drug sensitivity testing (DST) on OS-PDCs distinguishes between chemotherapy responders and non-responders and provides information on RTK sensitivity

Osteosarcoma patients are often heavily treated and have poor responses to chemotherapy. We conducted an ex vivo drug sensitivity study on patient-derived osteosarcoma (OS-PDCs) and Ewing sarcoma (ES-PDCs) cells from patients with recurrent or refractory disease with the primary objective to investigate the feasibility of the approach in identifying alternative drug treatments. The characteristics of the patients are presented in Table 1 and the characterization of the OS-PDCs and ES PDCs is shown in Table S1 and Supplementary Figure S1.

**Table 1:** Patient characteristics, diagnosis and treatment

ID	Gender	Age at diagnosis	Diagnosis	Localization	Met at diagn	Local recurrence	Met	Neoadj/adj Treatment	Treatment at relapse
SARC 16	M	12	Osteogenic OS	Dist diaphys femur	no	no	yes	Euramos	Surgery, Gem/Doc/Vin, Das, Paz
SARC 17	F	60	Teleangiectatic	Prox humerus	no	Yes	yes	Surgery	RT/thoracoscap amputation
SARC 20	F	47	Paraosteal sarc	Knee sin	no	No	yes	No adj treatment	Dox/Cis
SARC 26	F	19	Osteogenic OS	Th6-8	no	yes	yes	Euramos	SBRT 7Gyx5. Int, Pem, Surgery, RT, Eto, Pal( CDK4-6 amp)
SARC 27	F	37	ES/PNET	L5	no	yes	no	SSGIII, RT protons 60 GY	Gem/Doc, RT, Iri/Tem
SARC 30	M	13	Chondroblastic OS	Pelvic region	yes	yes	yes	Euramos	Hemipelvectomy, lung metastasis surgery, RT
SARC 32	M	23	Ewing sarcoma	Retrop.&intraspinal Th12-L2	no	yes	yes	Surgery, VIDE/ RT	Gem/Ifos, Paz, RT, Iri/Tem, Doc/Gem
SARC 33	M	26	Osteoblastic OS	Spina iliaca ant	yes	yes	yes	Euramos, single Dox	Eto/Ifo, Doc/Gem, RT, Paz, Thal
SARC 38	F	31	ES	Sacrum	no	yes	yes	SSG III, RT protons 60 GY	RT, Iri/Tem, Cab
SARC 39	M	13	ES	Thoracic wall, costa 6, dx	no	no	yes	EuroEwing 12, surgery, RT	Surgery, RT, Tem/Iri /Cel, proton RT, CTX stem cell transpl, Top/Cyc
SARC 41	M	18	Chondroblastic OS	Pelvic region dx	no	yes	yes	Euramos, no MTX, RT 70 GY	Eto/Ifo

VIDE: Vin/ Ifo/Dox/Eto Euramos: osteosarcoma (doxorubicin, cisplatin, methotrexate) EuroEwing 12: (Vincristine, Cyclophosphamide, Doxorubicin)

SSG III: Localized EWING (vincristine, doxorubicin, cyclophosphamide, actinomycin-D, ifosfamide, etoposide) Radiotherapy (RT) Doxorubicin (Dox) cabozantinib (Cab) celecoxib (Ceb) cisplatin (Cis) cyclophosphamide (Cyc) dasatinib (Das) docetaxel (Doc) etoposide (Eto) gemcitabine (Gem) ifosfamide (Ifo) interferon (Int) irinotecan (Iri) methotrexate (Mtx) palbociclib (Pal) pazopanib (Paz) pembrolizumab (Pem) regorafenib (Reg) temozolomide (Tem) thalidomide (Thal) topotecan (Top) vincristine (Vin) vinorelbine (Vino)

**Fig. 1:**
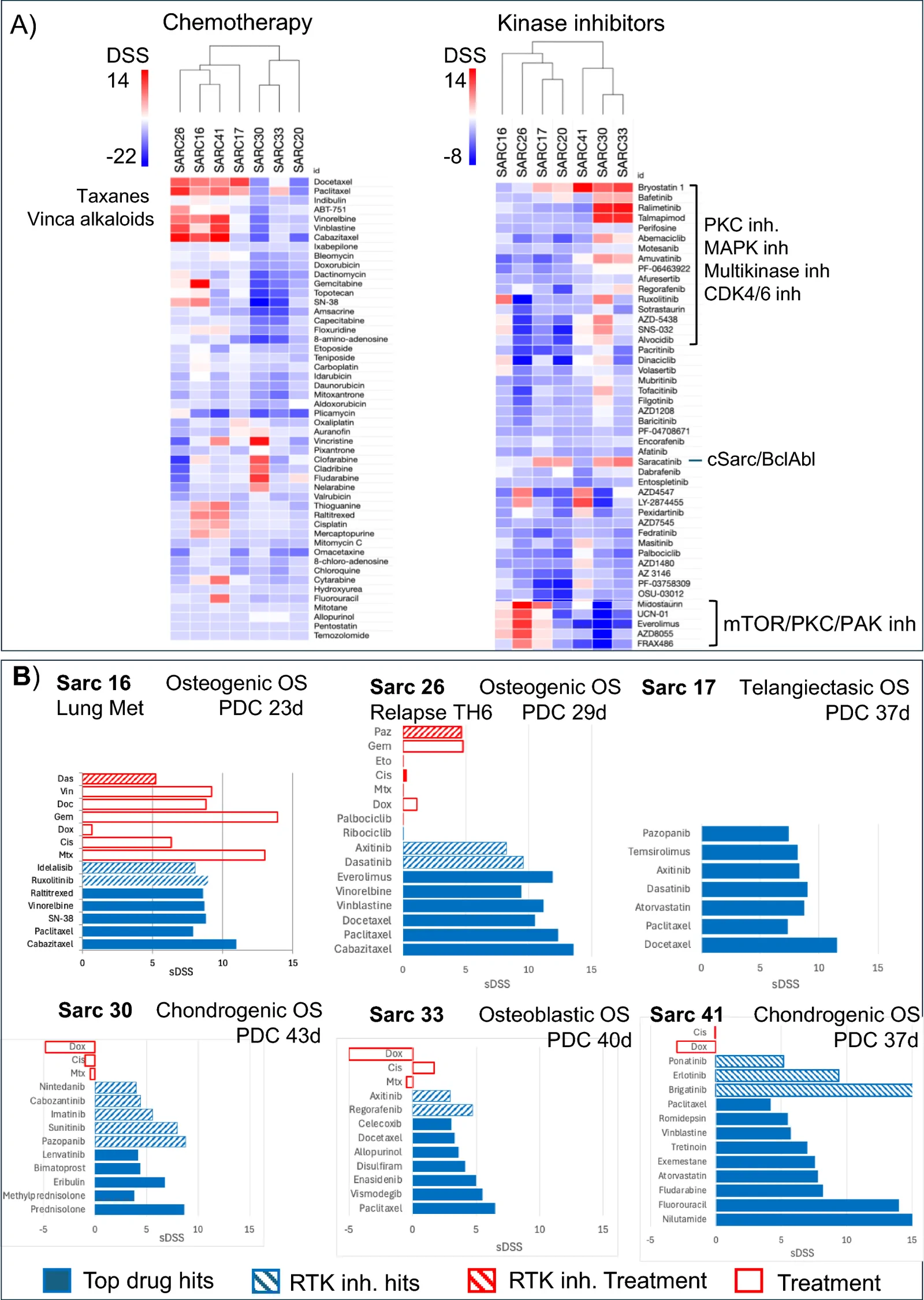
**A** Heat maps showing the hierarchical clustering of the drug sensitivity scores (DSS) of osteosarcomas PDCs to conventional chemotherapy drugs and kinase inhibitors. **B** Bar plots of the best drug hits for individual OS-PDCs. The patient ID, osteosarcoma subtype, biopsy site, and time from biopsy to drug screening in days (d) are indicated in each chart. Best drug hits (blue bars). Response to kinase inhibitors (striped bars) and actual patient treatment (red bars)

Cluster analysis of the drug sensitivity scores (DSS) of the seven OS-PDCs stratified the group into sensitive and non-sensitive to conventional chemotherapy in which chemotherapy resistance was associated with sensitivity to multikinase inhibitors (Fig. 1A). SARC 16, 17, and 26 were sensitive to specific cytostatic drugs such as taxanes (paclitaxel, docetaxel, and cabazitaxel) and vinca alkaloids (vincristine, vinorelbine, and vincristine) but showed poor response to TRK inhibitors. OS-PDCs from poor chemotherapy-response patients (SARC30 and SARC33), showed sensitivity to RTK inhibitors, specifically to p38/MAPK and Src/Lyn/Abl antagonists. One OS-PDC (SARC 20) was refractory to both chemotherapy and RTK inhibitors (Fig. 1A). The sensitivity to other drug classes such as hormone therapy, apoptosis, metabolism, or epigenetic modulators was variable among the OS-PDCs tested.

We then identified the best drug hits for each OS-PDC by subtracting the drug sensitivity scores of normal primary mesenchymal cells (muscle cells) from the OS-PDC values to obtain the selective drug sensitivity score (sDSS) (Fig. 1B). We found that the sDSS for each PDC correlated with the patient response to treatment and confirmed that PDCs with resistance to chemotherapy (SARC 30, SARC 33, SARC 41) had patient-specific sensitivity to multikinase inhibitors (Fig. 1B).

### Correlation between DST, genotype, and patient response to treatment

**Fig. 2:**
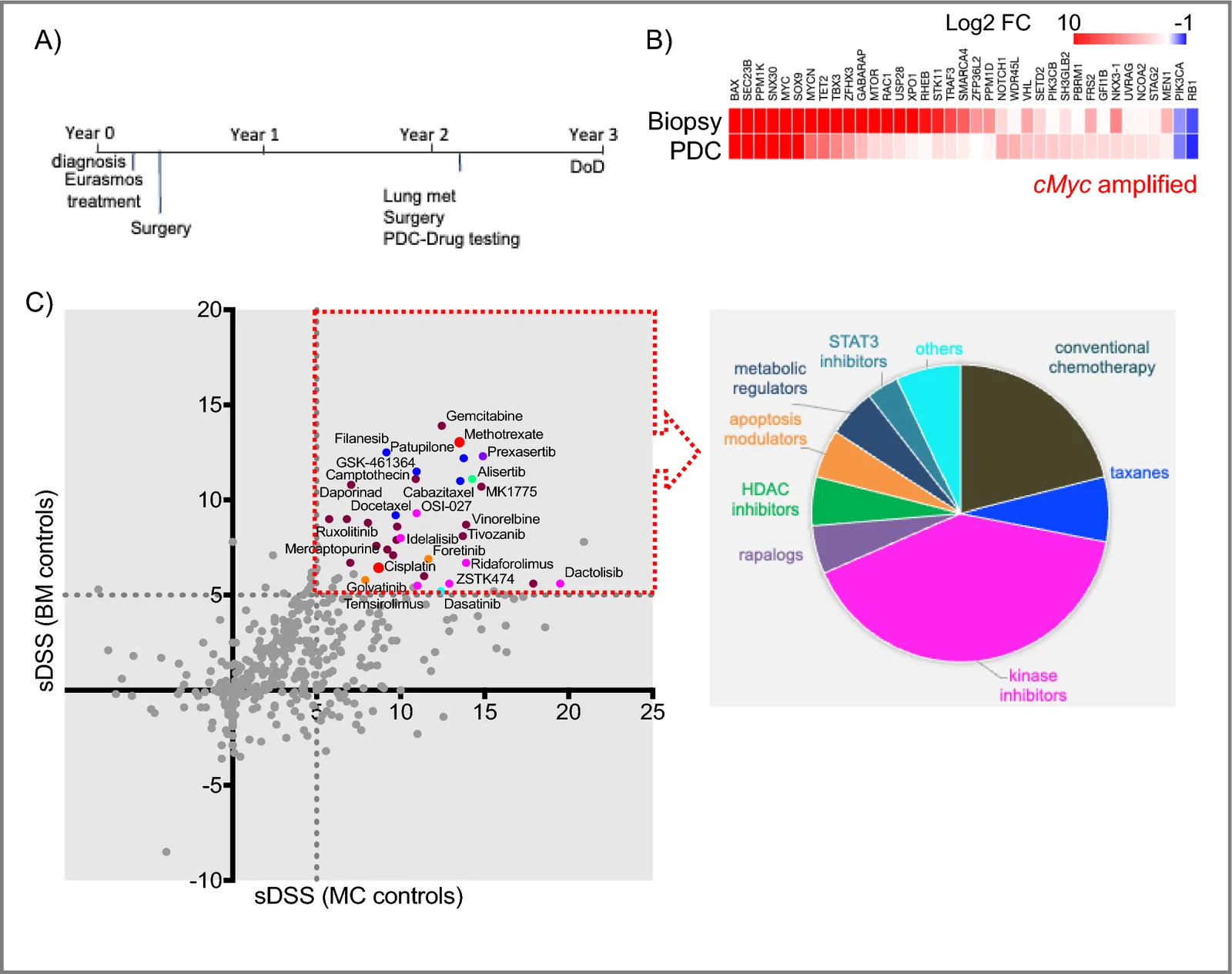
Drug sensitivity testing and genomic profile in a case of osteogenic sarcoma. **A** Time line of the patient clinical management. **B** Heat map showing cancer gene expression in the patient tumor (biopsy) and derived PDC generated by quantitative RT-PCR arrays and expressed as log2 fold change. Routine genomic analysis of the patient tumor showed amplifications of the *cMyc* gene. **C** Plot showing the selective drug sensitivity scores (sDSS) to all drugs in the library. sDSS was obtained in relation to normal bone marrow hematopoietic cells (BM controls) and in relation to healthy primary mesenchymal cells (MC controls). The best drug hits are found within the red rectangle and their drug class indicated in the respective color in the adjacent pie chart

Further correlation of the drug class sensitivity of each OS-PDC with the genetic characteristics of the patient tumor led to the identification of activated cellular pathways and target inhibitors for potential treatment. Here, we describe two such examples:

SARC 16. The patient, a 13-year-old boy was diagnosed with a osteoblastic osteosarcoma in the distal diaphysis of the femur. He underwent adjuvant chemotherapy according to the EURAMOS protocol consisting of methotrexate, doxorubicin and cisplatin, with good clinical response followed by surgery with local excision of the tumor, osteotomy and reconstruction with bone transplant. The patient developed lung metastasis 2 years after diagnosis. A surgical biopsy was obtained from a lung metastasis for ex vivo cell culture, and the PDC was established 23 days after surgery (Fig. 2A). Gene expression studies showed similarities in the transcriptional profile of both the tumor biopsy and the PDC with overexpression of c*Myc *and *MycN, Bax, PPM1K* and, *Sox9* (Fig. 2B). Consistently, the patient tumor carried an amplified *cMyc* gene. Drug sensitivity testing was performed on the PDC and confirmed the patient response to methotrexate and cisplatin treatment (Fig. 2C red dots). In addition, sensitivity was observed for multikinase inhibitors, rapalogs, HDAC inhibitors and taxanes (Fig. 2C, pie).

**Fig. 3:**
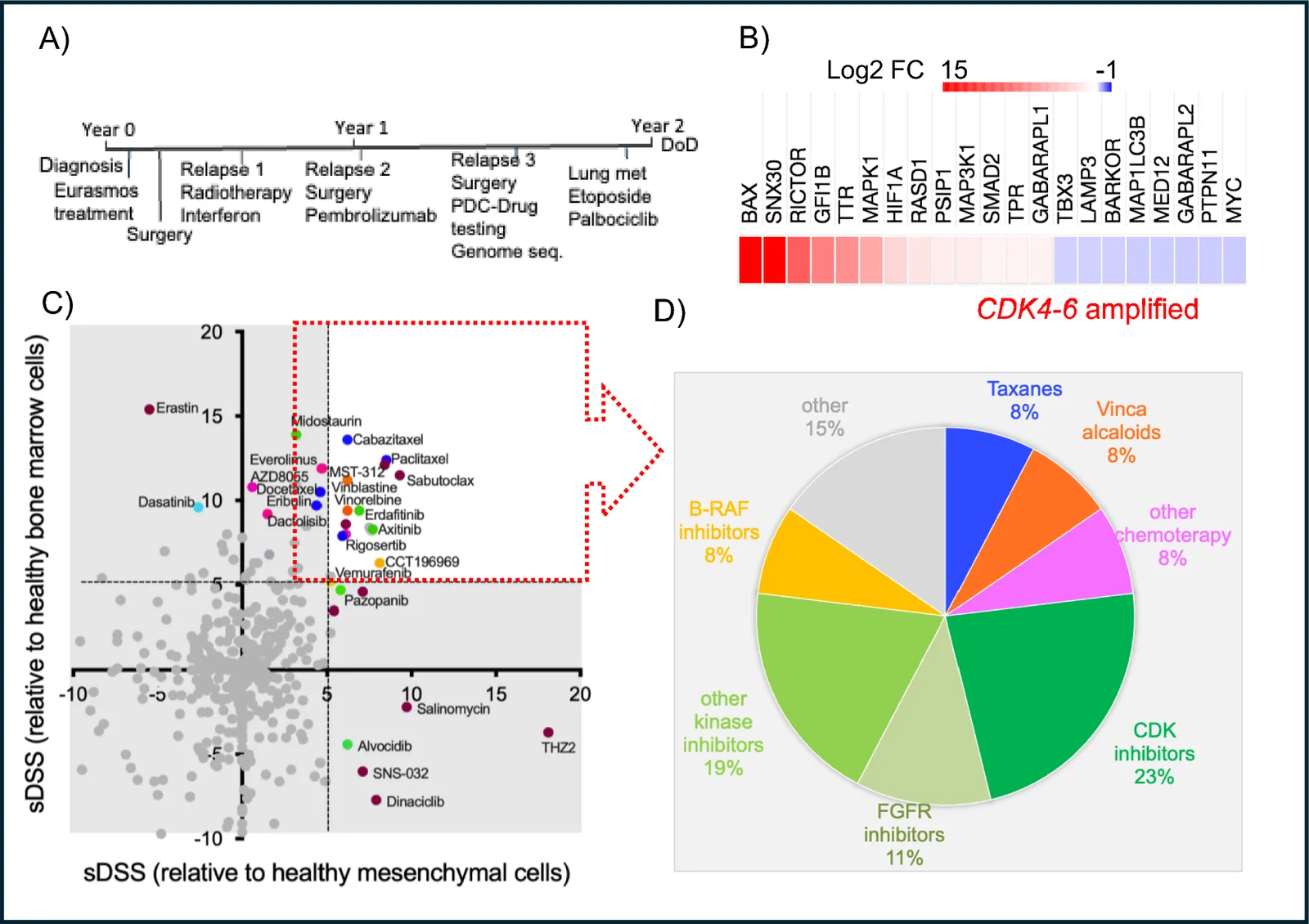
Drug sensitivity testing and genomic profile in a second case of osteogenic sarcoma. **A** Time line of the patient clinical management. **B** Heat map showing cancer gene expression in the PDC generated by quantitative RT-PCR arrays and expressed as log2 fold change. Routine Genomic characterization of the patient tumor showed *CDK4*and *CKK6* gene amplifications. **C** Plot showing the selective drug sensitivity sores (sDSS) to all drugs in the library. sDSS was obtained in relation to normal bone marrow hematopoietic cells (BM controls) and in relation to healthy primary mesenchymal cells (MC controls). The best drug hits are found within the red rectangle and their drug class indicated in the respective color in the adjacent pie chart

SARC 26. A 19-year-old female patient was diagnosed with a highly malignant osteogenic osteosarcoma, in Th 6–8. No fusion transcripts were found in the tumor biopsy. She underwent adjuvant treatment according to the EURAMOS protocol consisting of methotrexate, doxorubicin, and cisplatin with poor responses. The resection margins after surgery were intralesional leading to the first relapse in Th6 that was treated with stereotactic radiotherapy 7GYX5 with a total dose of 50 Gy, followed by interferon treatment. A 5-cm tumor relapse was diagnosed one year after interferon treatment in the Th6 and removed surgically. The patient received pembrolizumab as a third-line treatment with no clinical response and developed a third relapse that was surgically removed. Tumor samples from the third relapse and healthy muscle tissue were obtained after surgery for genome analysis and drug sensitivity testing. Consistent with the patient response to the EURAMOS regimen protocol, the drug sensitivity test showed poor responses to methotrexate, doxorubicin, and cisplatin and some response to taxanes and vinca alkaloids (Fig. 3C and D). Among the drug classes with cytotoxic activity, kinase inhibitors were overrepresented (Fig. 3C green). The patient developed lung metastases 2 years after diagnosis and was treated with etoposide. Genome sequence analysis showed CDK4–6 gains, and the patient initiated treatment with CDK inhibitor palbociclib; however, response could not be followed up due to the patient’s advanced disease.

Particularly relevant cases worth performing DST are those with poor clinical response to standard treatment protocols, as these cases show sensitivity to multikinase inhibitors, as shown in Fig. 1. SARC 33, a PDC obtained from an osteoblastic OS with synchronous lung metastases, displayed resistance to chemotherapy ex vivo that correlated with the patient’s poor clinical response to treatment and disease progression. The patient was further treated with pazopanib, but the treatment was interrupted due to pneumothorax. The derived  PDC showed sensitivity to axitinib and regorafenib. SARC 30 was obtained from chondroblastic OS developed in the pelvis with synchronous lung metastases. The patient received treatment according to the EURAMOS protocol with poor clinical response and progressive disease. Consistently, drug sensitivity testing of the OS-PDC showed resistance to doxorubicin, methotrexate, and cisplatin; however, it displayed sensitivity to several approved multikinase inhibitors. Interestingly, the PDC showed sensitivity to corticosteroids, suggesting potential involvement of an immuno-inflammatory component (Fig. 1B).

Although, with different etiology than primary osteosarcomas, we screened a radiation-induced telangiectatic OS (SARC 17). The patient did not accept chemotherapy; therefore, drug response correlation could not be performed. The PDC carried mutations in *RICTOR* and in the *p53* gene. Consistently, overexpression of mTOR and sensitivity to apoptosis inhibitors and rapalogs was observed (Supplementary Fig. 2).

### Ewing sarcoma PDC sensitivity to epigenetic drugs and morphological heterogeneity

**Fig. 4:**
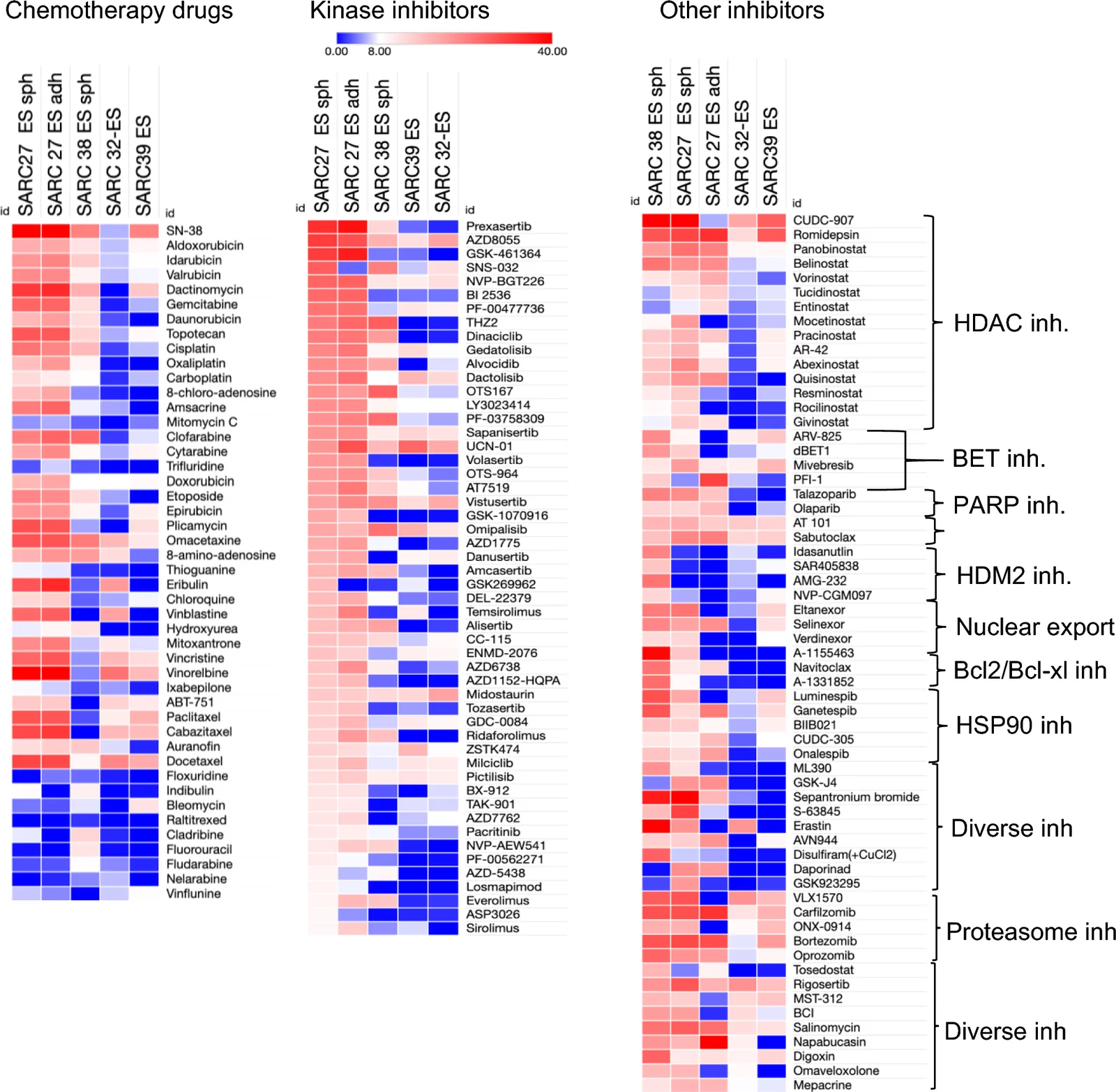
Heat maps showing the hierarchical clustering of the drug response of ES PDC to routine chemotherapy and kinase inhibitors and other targeted inhibitors. Heat maps were generated using the Morpheus software for data visualization and analysis

**Fig. 5:**
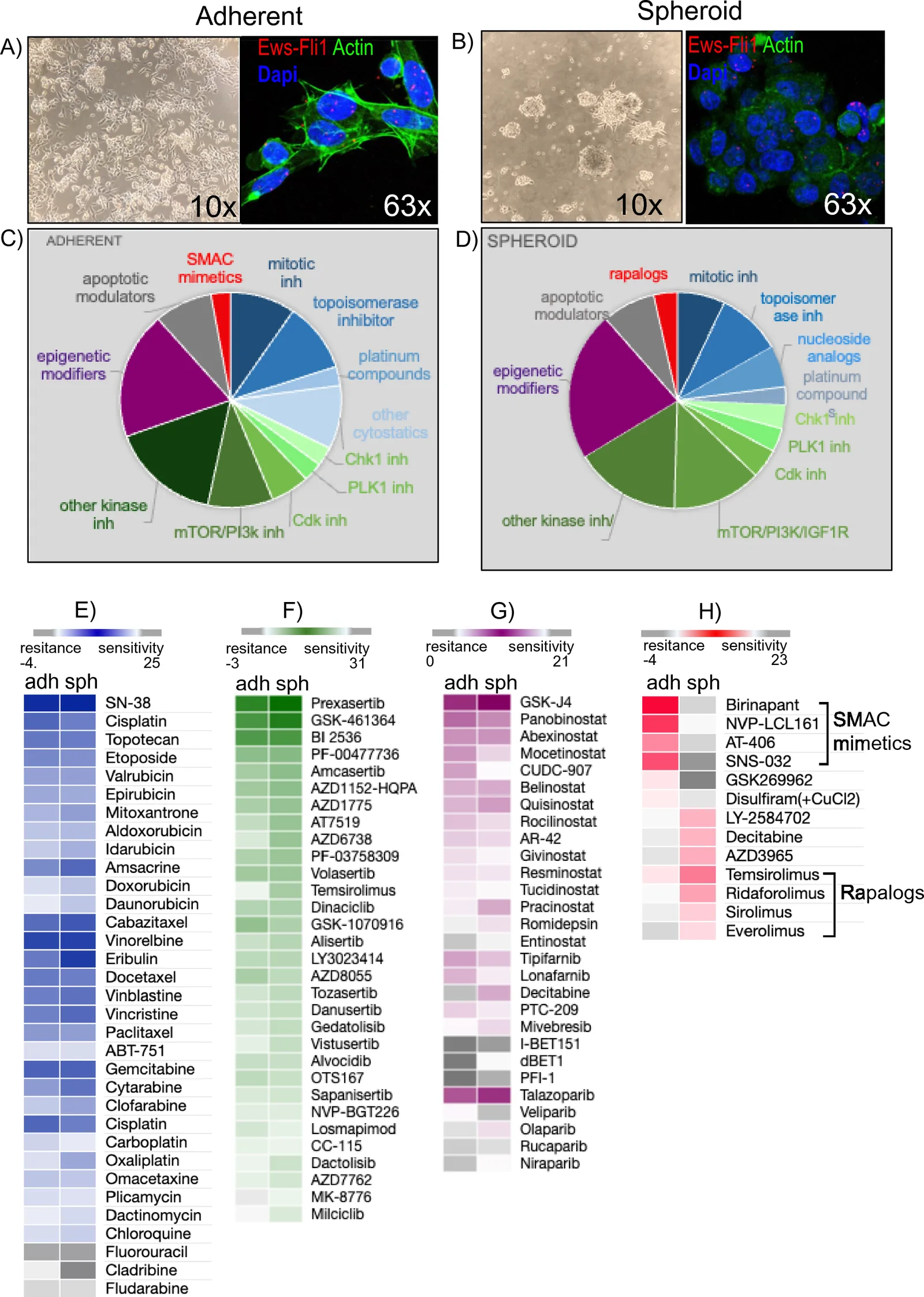
Drug sensitivity in adherent and spheroid populations from an ES PDC. **A** and **B** Transmission light microscopy and confocal immunofluorescent images of adherent (**A**) and spheroids (**B**). ES-PDC populations both expressing the Ews-Fli1 fusion gene (red dots) as determined by proximity ligation assay. Pie charts showing the response to specific drug classes (**C**, **D**). Heat maps comparing drug responses between adherent and spheroid populations to conventional chemotherapy (blue heat map, **E**), kinase inhibitors (green heat map, **F**), and epigenetic modifiers (purple heat map, **G**) are shown. Differential drug responses are shown (**H**)

Patient-derived Ewing’s sarcoma cells (ES-PDCs) were propagated from fine needle aspirations (FNA) or surgical biopsies. Four biopsy samples were successfully obtained for drug sensitivity testing. ES-PDCs displayed higher drug sensitivity scores (DSS) than OS-PDCs and often had sensitivity to epigenetic drugs (HDAC inhibitors) and proteasome/protein synthesis inhibitors (Fig. 4). In two cases (SARC 27 and SARC 38), the tumor cells spontaneously grew as two morphologically distinct populations, an adherent monolayer and a non-adherent spheroid population. One such case was SARC 27, derived from a patient who developed an EWSR1-FLI1 positive Ewing sarcoma in vertebrae L5. The patient was treated according to the SSG III protocol with poor clinical response and later received proton RT and stem cell harvest. The patient developed lung metastasis and local relapse, from which a biopsy was obtained for ex vivo culturing and drug screening. ES-PDCs growing as monolayers and spheroids spontaneously arose from the FNA biopsy after 2 weeks, both expressing the EWSR1-FI1 fusion protein (Fig. 5A and B). Drug sensitivity testing showed that both populations displayed similar responses to most of the drug classes, including cytostatic drugs, kinase inhibitors, epigenetic modifiers, and apoptosis modulators (Fig. 5C–G). Differential drug sensitivity was observed for the adherent ES-PDC population that, contrary to the spheroid type, was sensitive to SMAC mimetics, a class of small molecules that release the apoptosis blockade exerted by inhibitors of apoptosis proteins (IAPs) in tumor cells (Fig. 5H). The spheroid population, on the other hand, was sensitive to Rapalogs, to the hypomethylating agent decitabine, and to the monocarboxylate transporter (MCT1) inhibitor AZD3965.

These results show that ES-PDCs can be composed of heterogeneous populations with selective responses to oncological drugs.

## Discussion

In the present study, we performed drug sensitivity testing on patient-derived bone sarcomas to investigate the feasibility of this assay in identifying oncological drugs with potential therapeutic effects for patients with refractory osteosarcomas and Ewing sarcomas. The donor patients were already extensively treated and had progressive disease; therefore, the goal was not a curative treatment but a trial to test the potential benefit of introducing drug sensitivity testing for these patients.

The most important findings regarding drug sensitivity testing for osteosarcoma PDCs in the present study are: 1. the association of drug sensitivity ex vivo with patient response to routine treatment; 2. the ability to disclose patient tumor sensitivity to specific kinase inhibitors, particularly valuable for patients with refractory osteosarcoma; 3. the correlation of drug sensitivity with the genetics of the osteosarcoma subtype.

It is known that the genetic complexity of osteosarcomas combined with different histological subtypes generates wide heterogeneity among patient tumors; a drawback for genome-based precision oncology. In spite of the high genome variability, clinically actionable alterations are found in approximately 21% of osteosarcomas including amplifications of cMyc [22], CDK4, PDGFRA, and VEGFA genes [23], as well as recurrent aberrations of IGF signaling genes [9]. Consistently, we found drug response heterogeneity in our osteosarcoma PDC cohort; however, PDCs from patients with poor responses to chemotherapy retained sensitivity to patient-specific multikinase inhibitors targeting mTOR, PKC, MAPK, and CDK kinases. In fact, tyrosine kinase inhibitors (TKIs) are being introduced as a standard treatment modality for advanced and recurrent bone sarcomas, particularly osteosarcoma and Ewing sarcoma. Multi-target TKIs, such as cabozantinib, lenvatinib, regorafenib, and sorafenib are being investigated in several clinical trials with promising results [24].

Two multicenter studies are currently ongoing in Europe for OS and ES patients. The FOSTER CabOS study adds a 12 months maintenance therapy with cabozantinib after the first line standard therapy protocol. The trial is randomized against placebo to evaluate the effectiveness of cabozantinib. This new therapy aims to improve the survival in the OS patient group. The second study, rEECur clinical trial was set up to evaluate the four most used chemotherapy regimens with respect to efficacy and toxicity to patients with relapsed Ewing sarcoma, and to identify the best chemotherapy backbone to which novel targeted drugs could be added for treatment. High-dose ifosfamide was found to be the most effective, and the rEECur trial is now testing whether lenvatinib, a multiple tyrosine kinase inhibitor given together with chemotherapy is more effective than chemotherapy alone. On the light of the ongoing clinical trials, we believe that implementing drug sensitivity testing of osteosarcoma PDCs against approved kinase inhibitors could help in selecting the multikinase inhibitor with better likelihood of giving objective response for patients with relapsed osteosarcoma.

An important finding in the present study was the correlation between drug response and gene rearrangements for specific osteosarcoma subtypes. For example, we found sensitivity to CDK inhibitors in a PDC from osteogenic sarcoma with CDK4 amplifications (SARC26). The patient initiated treatment with the CDK4/6 kinase inhibitor palbociclib, unfortunately at an advanced stage of disease (Fig. 2). SARC16, an osteosarcoma with amplified *cMyc* gene and consequent overexpression of c-Myc and N-Myc in the tumor biopsy and derived PDC (Fig. 3). Sporadic amplifications of cMyc are found in children's osteosarcomas [22], so-called *c-Myc*-driven subtype that is associated with unfavorable prognosis [25, 26].

In a case of radiation-induced (telangiectatic) osteosarcoma, we found mutations in *TP53* and in the rapamycin-insensitive companion of mTOR (*RICTOR)* genes. *TP53* mutations predispose to the development of different cancer types including bone sarcomas [27]. The patient PDC overexpressed mTOR and displayed sensitivity to Rapalogs (Supplementary Fig. 2).

Tumor cell heterogeneity in ex vivo cultures was brought up in the present study by the finding that Ewing sarcomas sporadically will grow in vitro as morphologically different populations. This raises concerns whether these populations exist in the tumor of origin or if they are raised by adaptive clone selection in ex vivo cultures. In our study, two of four Ewing sarcoma primary cultures contained spheroid and adherent *Ews-Fli1* positive populations (Fig. 5 and Supplementary Fig. 1). Although both populations had similar drug sensitivity to most of the conventional chemotherapeutic drugs and kinase inhibitors, we identified selective drug classes for each population. The ES-PDC growing as spheroids was sensitive to rapalogs and to the monocarboxylate transporter 1 (MCT1) inhibitor AZD3965 indicating dependency on mTOR activation and lactate production for energy metabolism [28] The adherent population, on the other hand, displayed specific sensitivity towards the second mitochondrial activator of caspases (Smac) mimetics. These are small molecules that break down the apoptosis blockade of inhibitor of apoptosis proteins (IAPs). Smac mimetics are under evaluation in early clinical trials as monotherapy or in drug combinations [29].

Over the last decade, efforts from several research groups have been undertaken to establish efficient patient sarcoma cell models for ex vivo drug testing that reproduce with fidelity the phenotype and behavior of the tumor of origin and are able to predict patient response to anti-cancer drugs in a time frame that allows patient treatment. They include two-dimensional patient-derived cell cultures, tumor organoids [12, 20, 30–32] and patient-derived xenografts [13, 14, 33–36]. For sarcomas, it is particularly challenging to establish standardized PDC models because the histological heterogeneity of these tumors demands specific ex vivo culturing conditions. In addition, patients are heavily treated with preoperative neoadjuvant therapy, making it difficult to obtain viable biopsy samples [37].

Drug sensitivity testing is an effective approach for the rapid identification of new oncological drugs for patients with poor treatment options. It has shown good therapeutic response prediction and can be particularly useful in sarcoma cases with complex genomes such as osteosarcomas; when genome-driven approaches can be difficult to implement.

The use of approved drug libraries in the testing will facilitate prospective clinical studies in which the predictive power of drug screening on patient-derived tumor models can be properly evaluated.

## Supplementary Information

Below is the link to the electronic supplementary material.

Morphology and expression of EWS-FLI in patient-derived Ewing Sarcoma cells (ES-PDC). A) Illustration of the process from biopsy to Ewing Sarcoma PDC showing the presence of round small blue cells in the fine needle aspiration (FNA) leading to ES-PDC outgrowth in a representative case (20). B) Expression of the EWS-FLI1 fusion protein (red dots) determined by proximity ligation assay (PLA) in different ES-PDCs. Actin was visualized using phalloidin-Alexa Fluor 488 (green). The nucleus was visualised with DAPI. Supplementary file1 (PDF 165 KB)

PDC from a radiotherapy-induced telangiectatic OS. Gene mutation analysis identified a frame shift mutation on exon 4 of the p53 gene, and a nonsynonymous single nucleotide variant (SNV) in Rictor (A). Cancer gene expression in both the biopsy and PDC showed overexpression of MycN as well as mammalian target of Rapamycin (mTOR), apoptosis related genes(BAX and BCL2), and genes associated with autophagy (RB1CC1, SEC23, SEC 16) (B). Drug sensitivity testing showed poor response to conventional treatment (methotrexate, doxorubicin, cis-platin) but sensitivity to vinca alcaloids and taxanes (C), and to epigenetic modifiers such as histone deacetylase inhibitors (D).Consistent with the overexpression of BAX and BCL2 in the patient tumor and derived cultured cells (B), the PDC was sensitive to BCL2 inhibitors AT101, Sabutoclax,and to TP53 reactivators such as UMI-77, Idasanutlin and AMG232. Sensitivity to rapamacin analogs and mTOR/AKT kinase inhibitors was consistent with RICTOR mutations, which are associated with abnormal mTOR signaling. Supplementary file2 (PPTX 4429 KB)

Supplementary file3 (DOCX 20 KB)
