# Supplementary material for: Drug sensitivity testing of patient-derived bone sarcomas identifies selective kinase inhibitors for patients with refractory disease
Source: Clin Transl Oncol. 2026 Apr 13;28(10):4873–84. doi: 10.1007/s12094-026-04261-4 (PMC13601131; doi:10.1007/s12094-026-04261-4)

Supplementary Figure 1

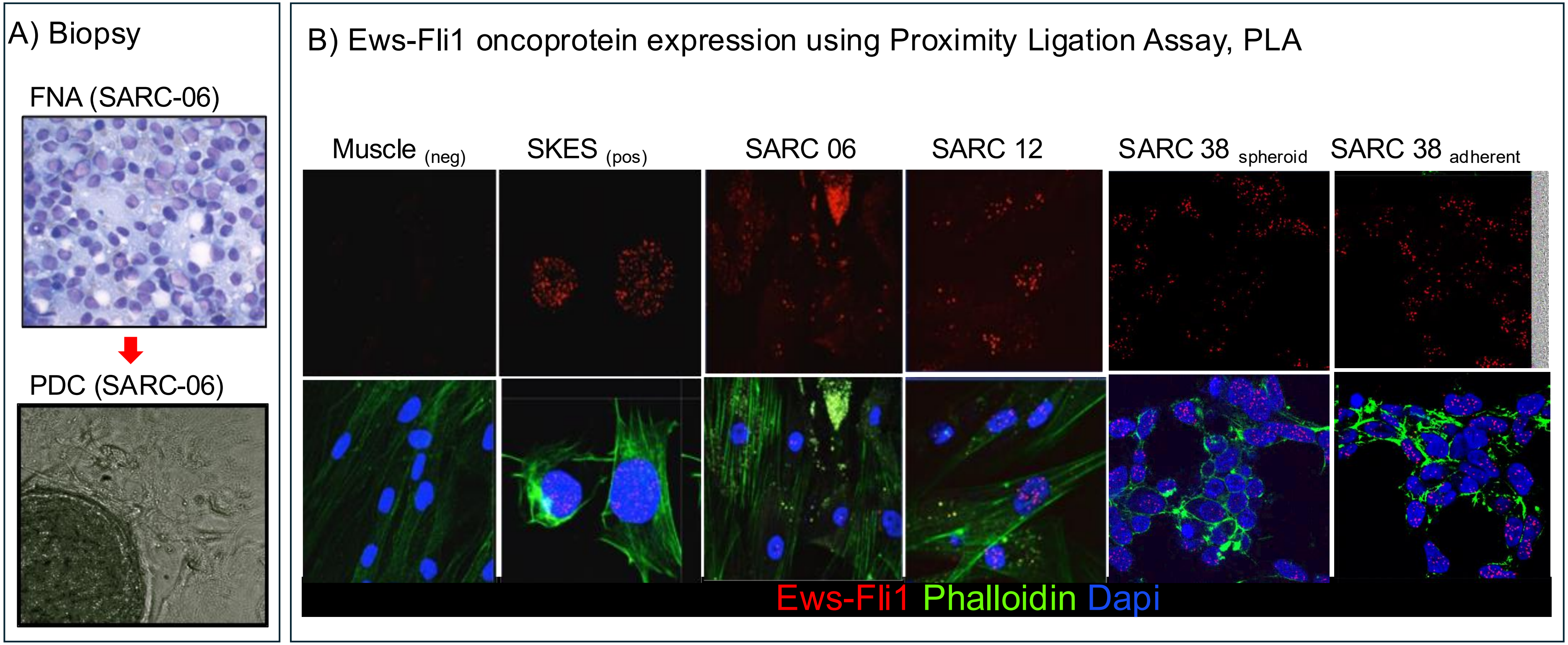

Supplement: Supplementary file 1 [file 12094_2026_4261_MOESM1_ESM.pdf]
