# Supplementary material for: Drug sensitivity testing of patient-derived bone sarcomas identifies selective kinase inhibitors for patients with refractory disease
Source: Clin Transl Oncol. 2026 Apr 13;28(10):4873–84. doi: 10.1007/s12094-026-04261-4 (PMC13601131; doi:10.1007/s12094-026-04261-4)
Supplement: Supplementary file 2 [file 12094_2026_4261_MOESM3_ESM.docx]

| **Supplementary Table 1.**  **Genetical markers of the bone sarcoma biopsies and derived PDC.** | | | | | |
| --- | --- | --- | --- | --- | --- |
| **ID** | **Sarcoma subtype** | **Biopsy type** | **PDC**  **time** | **Genomic alteration**  **biopsy** | **Genomic alteration**  **PDC** |
| SARC 16 | Osteogenic OS | Lung met.  Surgery | 23 d | No gene mutations found.  Overexpression (cMyc, Myc-N, Sox9, Bax). | No gene mutations found.  Overexpression (cMyc, Myc-N, Sox9, Bax) |
| SARC 17 | Teleangiectatic OS | Relapse  Surgery | 37 d | Gene mutation:  P53 exon 4 p.C143fs  RICTOR exon25 R821K | Gene mutation:  P53 exon 4 p.C143fs  RICTOR exon25 R821K |
| SARC-20 | Paraosteal OS | Subcut. relapse  Surgery | 61 d | Gene mutation:  CDKN2A exon 2 p.R58X  NF1 exon 17 p.P33L | Gene mutation:  CDKN2A exon 2 p.R58X  NF1 exon 17 p.P33L |
| SARC-26 | Osteogenic OS | Relapse TH6  Surgery | 29d | CDK 4/6 amplification | Overexpression:  Rictor and MAPK1 |
| SARC-30 | Chondroblastic OS | Pelvis  Surgery | 43d | Imaging  Histopathology | ND |
| SARC-33 | Osteoblastic OS | Open biopsy | 40d | Imaging  Histopathology | ND |
| SARC-41 | Chondroblastic OS | Pelvis  Surgery | 37d | Imaging  Histopathology | ND |
| SARC-27 | Ewing sarcoma  PNET | FNA | 45d | Ews/Fli1  (Q-RT-PCR) | Ews/Fli1  (PLA) |
| SARC-32 | Ewing Sarcoma | Core biopsy  intra spinal | 58d | Ews/Fli1  (Q-RT-PCR) | Ews/Fli1  (PLA) |
| SARC-38 | Ewing sarcoma | FNA  Rib | 75d | Ews/Fli1  (Q-RT-PCR)  ALK/ROS negative | Ews/Fli1  (PLA) |
| SARC-39 | Ewing Sarcoma | FNA  met/dura | 30d | Ews/Fli1  (Q-RT-PCR) | Ews/Fli1  (PLA) |

PDC time refers to the time from biopsy to PDC and drug screening. FNA: Fine needle aspiration

Gene mutations were determined by gene panel sequencing of 16 sarcoma-associated genes including *STAG2, CDKN2A, TP53, PTEN, NF1, BRAF, CTNNB1, SRC, RB1, CDH1, P14KA, RICTOR, EZH2, BCOR*, and *ARID1.* Gene expression/amplification was determined using a quantitative reverse-transcriptase.polymerase chain reaction (Q-RT-PCR) array containing 100 cancer-associated genes. Proximity ligation assay (PLA) was used to determine the expresion of the Ews-Fli1 oncoprotein in the Ewing sarcoma PDC using confocal immunofluorescence microscopy.
